# Discovery of Bioactive Compounds by the UIC-ICBG Drug Discovery Program in the 18 Years Since 1998

**DOI:** 10.3390/molecules21111448

**Published:** 2016-10-31

**Authors:** Hong-Jie Zhang, Wan-Fei Li, Harry H. S. Fong, Djaja Doel Soejarto

**Affiliations:** 1 School of Chinese Medicine, Hong Kong Baptist University, Hong Kong SAR, China; wanfeier@live.cn; 2 Department of Medicinal Chemistry and Pharmacognosy, College of Pharmacy, University of Illinois at Chicago, 833 South Wood Street, Chicago, IL 60612, USA; hfong@uic.edu (H.H.S.F.); dds@uic.edu (D.D.S.)

**Keywords:** UIC-ICBG, bioactive compounds, drug discovery, cancer, HIV, tuberculosis, malaria

## Abstract

The International Cooperative Biodiversity Groups (ICBG) Program based at the University of Illinois at Chicago (UIC) is a program aimed to address the interdependent issues of inventory and conservation of biodiversity, drug discovery and sustained economic growth in both developing and developed countries. It is an interdisciplinary program involving the extensive synergies and collaborative efforts of botanists, chemists and biologists in the countries of Vietnam, Laos and the USA. The UIC-ICBG drug discovery efforts over the past 18 years have resulted in the collection of a cumulative total of more than 5500 plant samples (representing more than 2000 species), that were evaluated for their potential biological effects against cancer, HIV, bird flu, tuberculosis and malaria. The bioassay-guided fractionation and separation of the bioactive plant leads resulted in the isolation of approximately 300 compounds of varying degrees of structural complexity and/or biological activity. The present paper summarizes the significant drug discovery achievements made by the UIC-ICBG team of multidisciplinary collaborators in the project over the period of 1998–2012 and the projects carried on in the subsequent years by involving the researchers in Hong Kong.

## 1. Introduction

Natural products have been a rich source for the discovery of therapeutic agents throughout the ages [1]. In contrast to the research philosophies of yesteryears, the research approach of the current project is based on the recognition that the potential for achievements and rewards is much greater when the process involves a collaboration among scientists in the countries rich in advanced biotechnology and the countries rich in biodiversity of natural resources. The ICBG (International Cooperative Biodiversity Groups) program was initiated in 1992 by the FIC (Fogarty International Center) of NIH (United States National Institutes of Health), NSF (National Science Foundation) and USDA (United States Department of Agriculture) [2], with the aims of fostering the cooperation between the industrialized countries and the nations of the developing world, to pursue the common goals of biodiversity conservation, drug discovery, and promoting economic growth of developing countries [2,3]. In response to the 1997 Request for Application (RFA: TW-98-001), a proposal with Dr. Djaja Doel D. Soejarto as PI (project leader) was put forward to establish and implement an ICBG project based at UIC (University of Illinois at Chicago) [4,5]. This ICBG project is known as “Studies on Biodiversity of Vietnam and Laos”, or UIC-ICBG in abbreviation, had been funded continuously by FIC from 1998 to 2012. Further, the legacies of the project have been carried on through the subsequent collaborative efforts involving researchers in Hong Kong.

The objectives of this ICBG program included: (i) integrated effort of biodiversity inventory and conservation at Cuc Phuong National Park (CPNP) in Vietnam, that will include the preparation of a Manual for taxonomic identification of the flowering plants in the park, the establishment of a Threatened Plants Rescue Center, the implementation of a conservation education program, and the transfer of GIS-based biodiversity assessment technology to Vietnam; (ii) integrated study of medicinal plants of Laos through the strengthening of the Lao Medicinal Plant Database and through a comparative ethnobotany mapping project in selected ecogeographic zones in Laos; (iii) collection of plant samples at CPNP and in Laos as an integral part of the UIC-ICBG drug discovery effort; (iv) drug discovery and development of anti-HIV, anti-bird flu, anti-malaria, anti-TB , and cancer chemoptherapeutic and chemopreventive agents from plants of Vietnam and Laos; (v) setting up the infrastructure and the human resource for the preservation of traditional knowledge in the uses of plants in primary health care of local communities through the establishment of new and the upgrading of existing ethnomedical gardens; and, lastly; (vi) strengthening the capacity (institutional infrastructure and human resources) of host institutions in Vietnam and Laos, in higher level of expertise, to undertake research in biodiversity study and conservation, ethnobotany, and plant-based drug discovery far into the future, beyond ICBG. 

More than 60 scientists and scholars in different disciplines of the life sciences have participated in the project, and the program has supported eight Master or Ph.D. degree students. Approximately 5500 plant samples including about 1100 ethnobotany based samples were collected from Vietnam and Laos [2,4,5,6].

The drug discovery program encompassed biological evaluation and phytochemical study of the plant extracts is one of the main components of the ICBG project based at the UIC. The candidate plants were selected for collection based on two approaches [2]. One is a biodiversity-based collection of plant samples, also referred as a “random” collection, with a goal to maximize the taxonomic diversity. The other one is an ethnobotany-based approach, whereby plants were collected based on the historical use of medicinal plants, especially those which have been used for the target diseases of the ICBG program [6]. Of the cumulative total of more than 5500 plant samples (representing more than 2000 species) that were collected in the two countries, 1901 have been evaluated for anti-HIV, 704 for cancer chemoprevention (i.e., quinone reductase, Cox-1, Cox-2, aromatase, luciferase-ARE and luciferase NF-κB), 2786 for cytotoxicity, 1848 for HL-60 differentiation, 2268 for antimalarial, and 2066 for anti-TB (tuberculosis) activities. More recently, a new viral entry inhibition assay was introduced for the evaluation of anti-bird flu and anti-HIV effects. Using this evaluation system, the 1859 previously untested plant extracts were evaluated, leading to the identification of six anti-bird flu and 11 anti-HIV plant leads [7]. One of the anti-HIV plant extracts, *Justicia gendarussa* showed potent inhibition activity against viral entry with an IC_50_ value of 0.04 µg/mL [8].

Of particular relevance to this review concerns the record of the bioassay-guided natural products chemistry accomplishments achieved in the UIC-ICBG project. Since the projects’ inception in 1998, we have isolated approximately 300 compounds of varying degrees of structural complexity, novelty and/or biological activity (anti-HIV, anti-malaria, anti-TB, or anticancer) from more than 30 active plant leads at UIC. The isolates include a series of anti-HIV and anticancer phytochemicals belonging to two new and one little known carbon skeletons, as well as potent antimalarial agents. The bioactive and/or novel isolates obtained are described below.

## 2. Active Agents

Natural products have long been the main resource in the search for potential lead compounds for the development of a large variety of therapeutic drugs including anticancer and antiviral agents [1]. One of the research missions of the UIC-ICBG was to discover new/novel molecules from tropical plant materials. The discovered bioactive compounds may serve as promising leads for future drug development. During the project, the plant extracts, the separated fractions, the purified compounds, and the synthesized derivatives were subjected to bioactivity evaluation in various in vitro assays. The disease targets of this drug discovery program mainly focused on the search for therapeutic agents that targeted cancer, HIV (human immunodeficiency virus), TB and malaria.

### 2.1. Anticancer Active Agents

**Figure 1:**
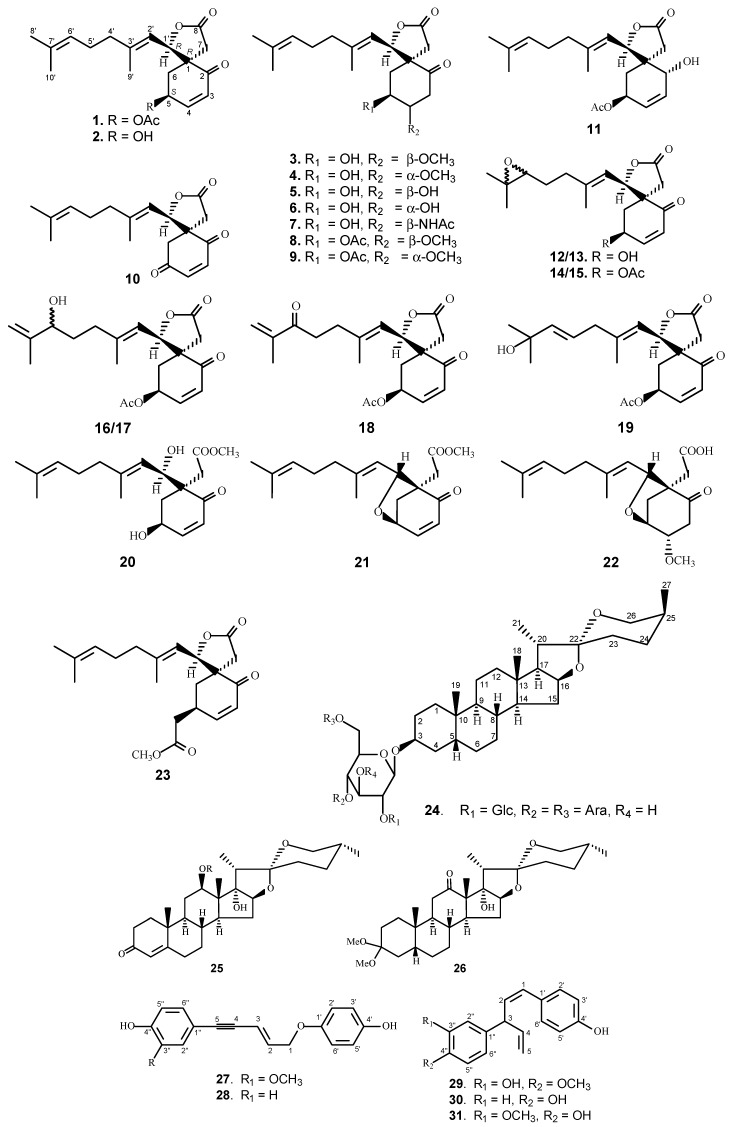

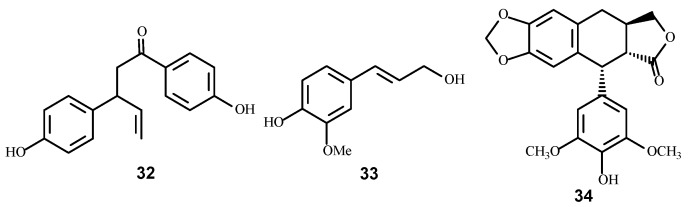
The chemical structures of compounds **1**–**34**.

A specific goal of the UIC-ICBG program is to discover potential antitumor candidates. In accordance with this widely-held program, from a total of 2786 extracts of terrestrial plants collected in Vietnam and Laos evaluated for cytotoxic activity in a panel of cell lines consisting of KB (human cervial carcinoma, a Hela derivative previously referred to as oral epidermis), Col-2 (human colon carcinoma), LNCaP (hormone-dependent human prostate cancer), Lu-1 (human lung cancer), MCF-7 (human breast carcinoma), hTERT (human telomerase reverse transcriptase immortalized), and HUVEC (primary human umbilical vein endothelial) cells, 327 samples were deemed active in the initial screen. Subsequent chemical study of 22 rank ordered plant extracts derived from 17 species led to the isolation of 32 active compounds including 15 new molecules (Figure 1 and Table 1) [9,10,11]. Among these active compounds, members of a rare C18 carbon compounds (**1**–**22**), designated as “miliusanes”, were of special interest. The anticancer activity of the miliusanes has been evaluated in the murine hollow-fiber in vivo assay, and three miliusanes (**1**–**3**) were further evaluated in the 60-cell line system by the National Cancer Institute (NIH, USA).

Among the selected plant samples for drug discovery phytochemical study, a dichloromethane extract of *Miliusa sinensis* Finet & Gagnep. (Annonaceae) collected in the Cuc Phuong National Park (Nho Quan District, Ninh Binh Province, Vietnam) exhibited cytotoxicity against KB cells with an IC_50_ value (concentration required to inhibit cell growth by 50%) of 2.0 μg/mL during initial bioassay [9]. The Bioassay-guided fractionation of the leaves, twigs and flowers of *M. sinensis* led to the isolation of a cluster of novel anticancer agents belonging to a rare skeletal group of C-18 terpenes (miliusanes). Of the 22 miliusane isolates, 20 are new molecules. Nine of these compounds (**1**–**3**, **5**, **8**, **9**, **18**, **20** and **21**) demonstrated significant cytotoxic activity in a panel of cell lines [9]. It has been noted that the presence of different functional groups significantly affected the cytotoxicity of this group of compounds (miliusanes I (**3**) vs. V (**7**)). Cytotoxic potency was also affected markedly by configurational differences in the functional groups (miliusanes I (**3**) vs. miliusanes III (**5**) vs. IV (**6**)). The epimers of 4β-hydroxyl group showed much better cell killing activity than their respective 4α-epimers. Interestingly, the investigators have observed that the cytotoxicity was not reduced to any extent when the γ-lactone ring was opened (miliusanes XVIII (**20**) and XIX (**21**)). In an attempt to improve the bioactivity of the miliusanes, 42 derivatives were prepared by esterification of the C-5 hydroxyl group of **1**. Although only a few of the derivatives showed equivalent or slightly better cytotoxicity, the derived methoxyacetyl-miliusol did demonstrate selectivity in a panel of cancer cell lines. MCF-7 was observed to be 9–15 times more susceptible to methoxyacetyl-miliusol (**23**) than the other four cell lines [9].

*Asparagus cochinchinensis* (Lourerio) Merrill (Asparagaceae) has long been used to treat chronic fever in Laos, China and Korea [10,12]. Bioassay-directed fractionation of the dried roots of *A. cochinchinensis* led to the isolation of five new (**24**–**27** and **29**) and five known compounds (**28** and **30**–**33**). Among the isolates, compounds (**24**, **29** and **31**) demonstrated moderate cytotoxicity against KB, Col-2, LNCaP, Lu-1, and HUVEC cell lines, with IC_50_ values ranging from 4 to 12 μg/mL (4–58 µM), while compounds **25**, **27** and **28** showed cytotoxicity toward KB cells only. 

*Bursera tonkinensis* Guillaum. (Burseraceae) is another plant that showed cytotoxic potential in the UIC-ICBG program. The CH_2_Cl_2_ extract from the roots of *B. tonkinensis* collected in Cuc Phuong National Park exhibited cytotoxicity against KB cells with an IC_50_ value of 4.1 μg/mL. As a result of a phytochemical study, 12 compounds were isolated from the roots of *B. tonkinensis*, including burselignan, bursephenylpropane, and burseneolignan [11]. Among the isolates, 4′-demethyldesoxypodophyllotoxin (**34**) showed potent cytotoxic activity against KB, Col-2 and LNCaP cell lines with IC_50_ values of around 10 ng/mL [11].

### 2.2. Anti-HIV Active Agents

**Figure 2:**
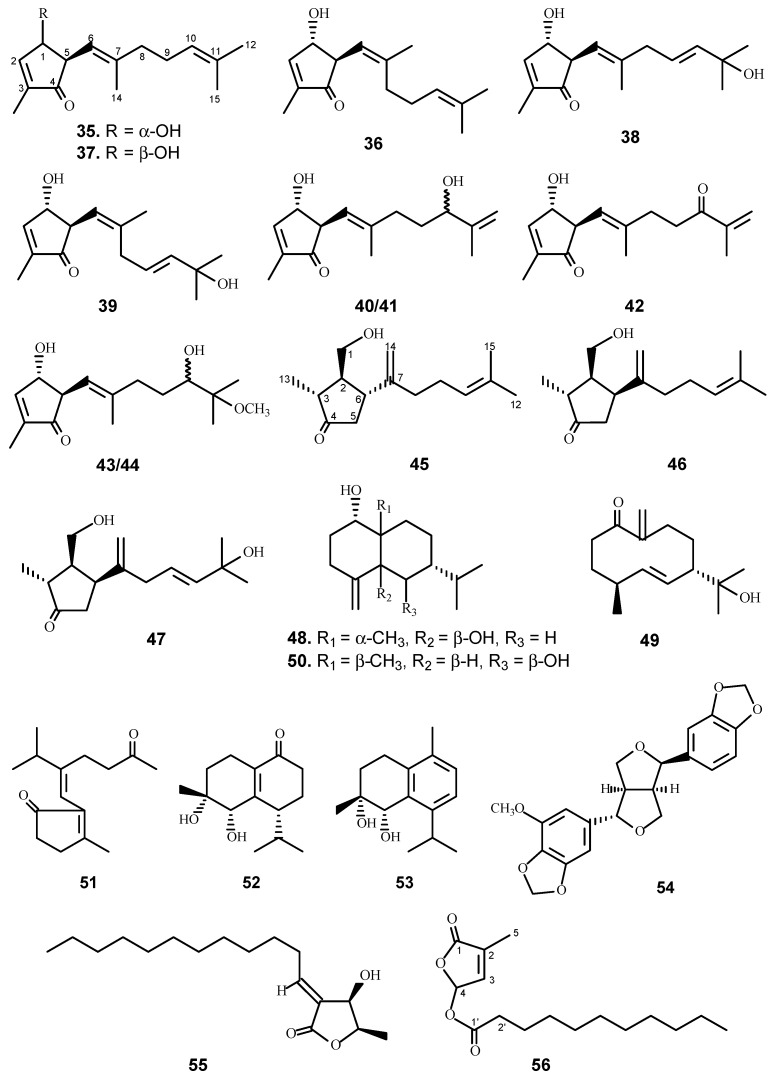

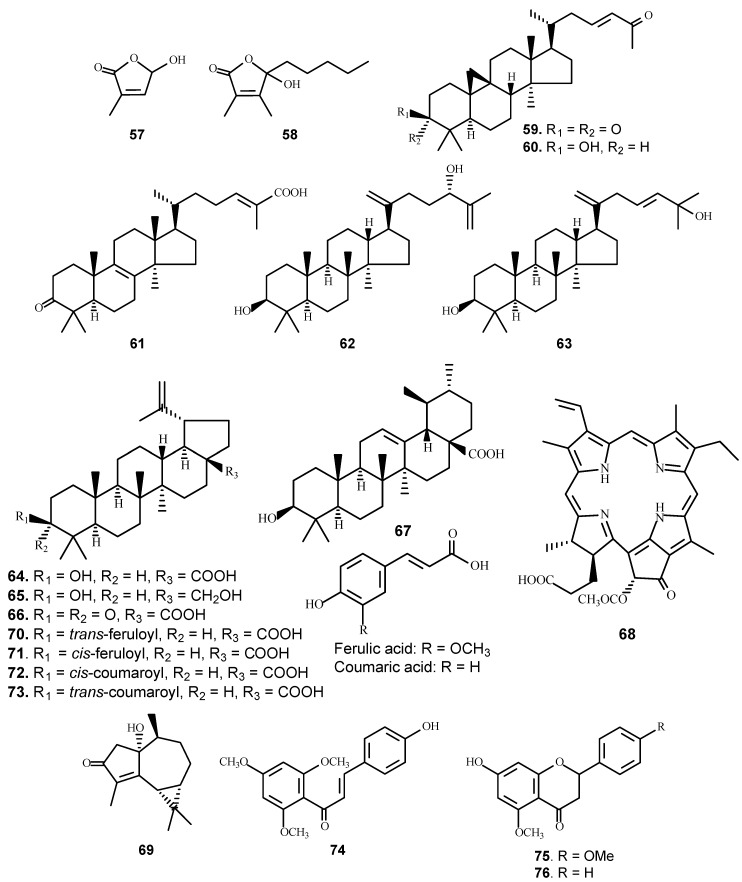
The chemical structures of compounds **35**–**76**.

Another goal of the UIC-ICBG program is to discover new/novel compounds against HIV. Two protocols were performed to evaluate the anti-HIV activity. The first one was quantitated using GFP (green fluorescent protein) reporter cell lines HOG.R5 [13]. Briefly, a reporter cell line for quantitating HIV-1 replication was developed using HOS (human osteosarcoma) cells rendered susceptible to HIV-1 infection by the transfection of genes for CD4 and CCR5, the co-receptor utilized by macrophage-tropic (R5) HIV-1 isolates. The other protocol used was so-called “One-Stone-Two-Birds” evaluation system, a more concise, safe and efficient assay to identify anti-flu (entry) and anti-HIV (replication) activities [7]. A total of 1901 plant extracts has been screened using the HOG.R5 reporter cell line, and an additional 1859 extracts were evaluated in the antiviral entry assay system (“One-Stone-Two-Birds” evaluation system) for anti-HIV activity. Chemical study of 32 prioritized active extracts led to the isolation of 42 bioactive compounds (Table 2 and Figure 2) including 24 new molecules [14,15,16,17,18,19,20,21,22]. 

Among the plants investigated, the chloroform extract of the leaves and twigs of *Litsea verticillata* Hance (Lauraceae) collected from Cuc Phuong National Park area [4] displayed significant inhibition activity against HIV-1 in a concentration of 20 μg/mL with minimal toxicity (90% cell viability). Anti-HIV bioassay-directed fractionation of *L. verticillata* led to the isolation of 24 anti-HIV compounds of a number of different skeletal types, including more than 10 different classes of sesquiterpenes (**35**–**53**), lignans (**54**) and butenolides (**55**–**58**) [14,15,16,17,20]. The sesquiterpenes belong to 13 different skeletal types, including two new sesquiterpene carbon skeletons. One of the skeletons was a novel one that the investigators designated as litseane [14], with a second one being given the name of isolitseane [18]. Ten litseanes (**35**–**44**) and three isolitseanes were isolated (**45**–**47**) [14,20], with all of the natural litseanes and one isolitseane showing anti-HIV activity with IC_50_ values ranging from 8–58 µM in the HOG.R5 system. Total synthesis of litseane compounds have since been reported by two independent research groups. The Vassilikogiannakis group achieved the total synthesis of litseaverticillols A–H by means of a biomimetic sequence of transformations initiated by a [4 + 2] reaction cascade and involving singlet oxygen (^1^O_2_) as the key step [23,24,25,26,27]. While the Kuwahara group accomplished the first enantioselective total synthesis of the (1*R*, 5*S*)-stereoisomer of litseaverticillols A and B by employing the Evans asymmetric aldol reaction and a microwave-promoted cyclization of a stannylated thiol ester intermediate as the C-C bond-forming steps [28,29]. A French group conducted synthetic study of the isolitseanes and analogues [30]. 

During the initial bioassay evaluation, a chloroform-soluble extract prepared from the twigs and leaves of *Vatica cinerea* King (Dipterocarpaceae) collected from the Cuc Phuong National Park was shown to inhibit HIV-1 replication by 86% with no cellular toxicity at 20 μg/mL. Accordingly, bioassay-guided separation led to the isolation of 11 active compounds (**59**–**69**), including a new triterpene (**59**) [19]. The majority of the triterpenes, sesquiterpene, 1-hydroxycyclocolorenone, and pheophorbide a isolated from this plant showed anti-HIV activity, with the chlorophyll being the most active, demonstrating an IC_50_ value of 1.5 μg/mL (2.5 μM), while being completely devoid of toxicity up to a concentration of 20 μg/mL (33.8 μM). 

Three new betulinic acid derivatives (**70**–**72**) and three known triterpenes (**64**, **67** and **73**) were isolated from the leaves and twigs of *Strychnos vanprukii* Craib. All of them showed moderate anti-HIV activity with IC_50_ values ranging from 5 to 11 μM [21]. 

*Vitex leptobotrys* H. Hallier f. (Lamiaceae) was selected for further bioassay-directed fractionation based on the result that the chloroform extract of the leaves and twigs inhibited HIV by 66% at a concentration of 20 μg/mL without showing any toxicity to the host cells at the same concentration [22]. Accordingly, 13 compounds were isolated and identified from this plant, and three of them (**74**–**76**) were found to have anti-HIV activity.

### 2.3. Anti-TB Active Agents

**Figure 3:**
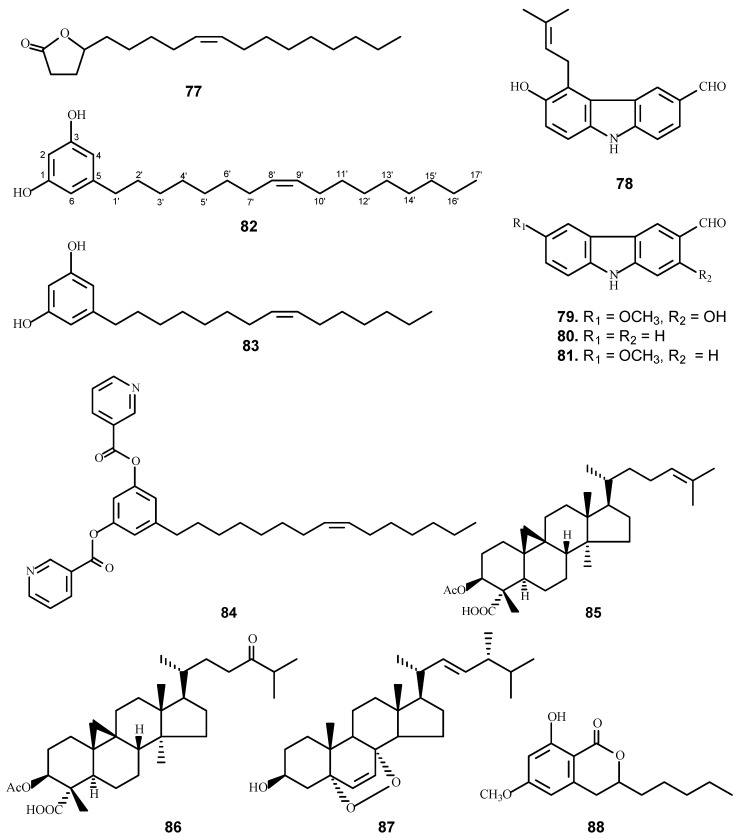
The chemical structures of compounds **77**–**88**.

Tuberculosis (TB) is a disease caused by *Mycobacterium tuberculosis* (H_37_Rv), which affects approximately 3 million annual deaths in the 1990s, and the TB mortality has fallen 47% since 1990, with nearly all of that improvement taking place since 2000 [31,32]. As part of the UIC-ICBG project, extracts were primarily screened against *M. tuberculosis* H37Rv using the microplate Alamar blue assay (MABA) [33] and low-oxygen recovery assay (LORA) [34]. The MIC is defined as the lowest concentration effecting a reduction in fluorescence or luminescence of 90% with respect to untreated controls. Accordingly, five of the most active plants selected from the evaluated 2066 plant extracts were carried out for bioassay-guided fractionation, which led to the isolation of 11 active compounds (Table 3 and Figure 3) including six new molecules [35,36,37,38]. 

Anti-TB bioassay-directed fractionation of the extract of the stem bark of *Micromelum hirsutum* Merr. (Rutaceae) collected from the Cuc Phuong National Park (Vietnam) led to the isolation of six carbazole alkaloids, as well as the γ-lactone derivative of oleic acid [35]. Five of the isolates (**77**–**81**) showed anti-TB activity. Among the active compounds, a fatty acid lactone, (−) *Z*-9-octadecen-4-olid (**77**) showed promising in vitro anti-TB activity with a MIC value (the drug concentration effecting an inhibition of 90% or greater) of 1.5 µg/mL with a selectivity index (SI) of 63 based on its cytotoxicity against the VERO cells, and exhibited activity against the Erdman strain of *M. tuberculosis* in a J774 mouse macrophage model with an EC_90_ value of 5.6 μg/mL) [35]. This suggested (−) *Z*-9-octadecen-4-olid might be a potential new anti-TB agent and worthy of further study. 

*Ardisia gigantifolia* Stapf (Primulaceae) has been used as a medicinal plant to eliminate blood stasis, disperse swelling, improve blood circulation, and also as an analgesic [39]. Antitubercular (anti-TB)-guided isolation of the CHCl_3_ extract of the leaves and stems of this plant led to the isolation of two active 5-alkylresorcinols (**82** and **83**) [37]. Fifteen (15) derivatives were further synthesized based on the two natural compounds to improve the bioactivity against tuberculosis. Only one compound (**84**) was found to show slightly improved anti-TB activity; since the compound contains nitrogen, it can be made in a water soluble form by preparing it as a salt compound, hence, worthy for further study as a novel anti-TB agent. 

A plant extract (*Radermachera boniana* Dop, Bignoniaceae), collected from the Cuc Phuong National Park, was found to inhibit the growth of *M. tuberculosis* H_37_Rv with a MIC value of 78 μg/mL. Bioassay-directed fractionation of the plant led to the isolation and structural elucidation of three new triterpenoids together with six known compounds. Among the isolates, bonianic acids A (**85**) and B (**86**) and ergosterol peroxide (**87**) exhibited significant activity against *M. tuberculosis* H_37_Rv strain [36]. 

Two new glucosides and seven known compounds were isolated from the stem bark of *Xylosma longifolia* (Flacourtiaceae), and the isolate 8-hydroxy-6-methoxy-pentylisocoumarin (**88**) exhibited an MIC value of 40.5 μg/mL against *M. tuberculosis* [38].

### 2.4. Antimalarial Active Agents

**Figure 4:**
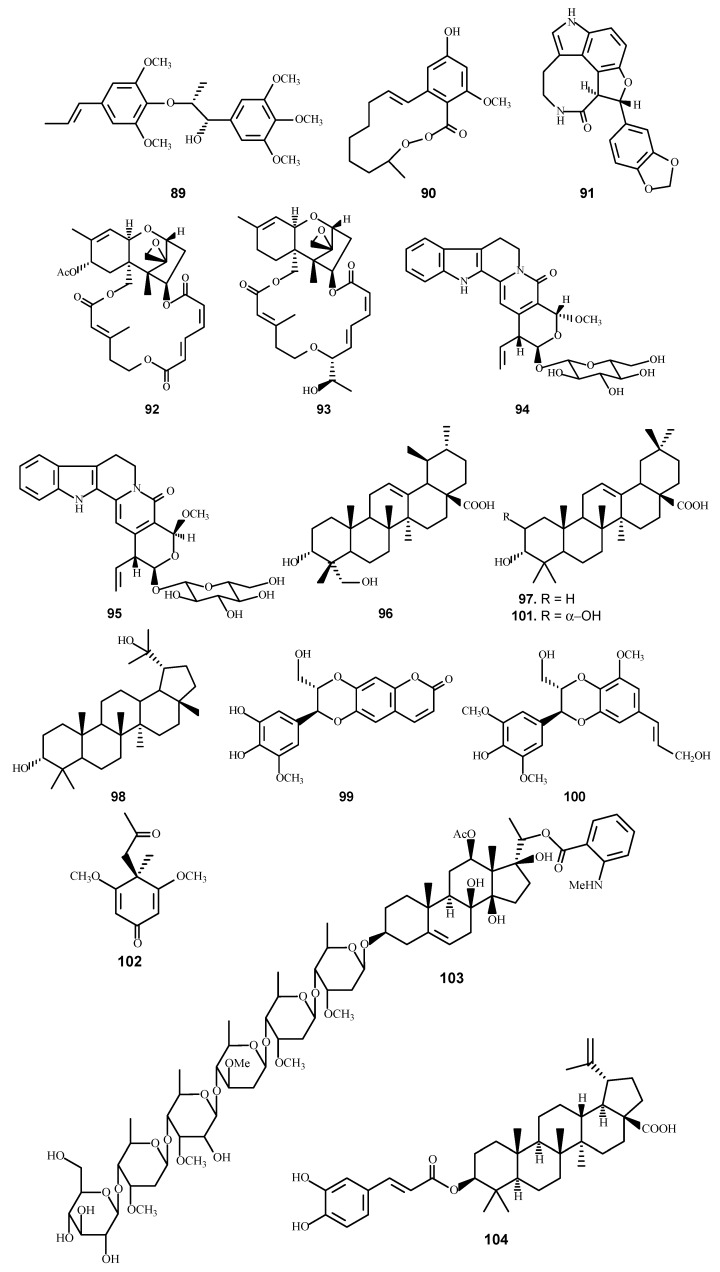

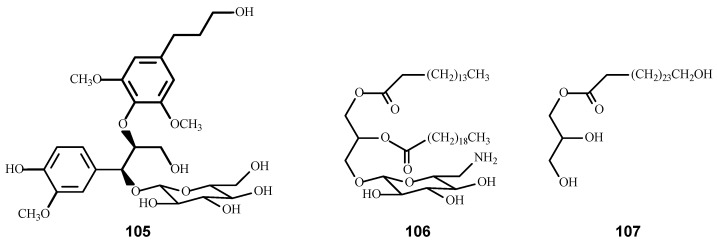
The chemical structures of compounds **89**–**107**.

The study of plant species of South Asia as an important source for the discovery of antimalarial agents is also a major objective of the UIC-ICBG project. Aside from tuberculosis and AIDS, malaria is a tropical disease that affects about 40% of people in the world [40,41]. Hence, the discovery of novel antimalarial agents is very much needed. Antimalarial assays of plant extracts and pure compounds were conducted with cultured chloroquine-sensitive parasites, using clone D6 derived from CDC Sierra Leone and chloroquine-resistance clone W2 derived from CDC Indochina [42]. During this project, a total of 2268 plant extracts were evaluated for antimalarial activity against *Plasmodium falciparum* clones D6 and W2. From the active extracts, 19 active compounds (Table 4 and Figure 4), with 12 being novel, were obtained [43,44,45,46,47,48,49,50]. 

Through collaborations established with several institutes in Vietnam, Laos, and the United States, the leave/stem extracts of *Rhaphidophora decursiva* (Roxb.) Schott (Araceae), found in the Cuc Phuong National Park, were shown to be active against both the D6 and W2 clones of *P. falciparum* with IC_50_ values less than 4 μg/mL [48]. Bioassay-directed fractionation led to the isolation of 18 compounds from the dried leaves and stems of *R. decursiva*, six (**89**–**93**) of them possessed antimalarial activity [43,47,48].

Among the active compounds were two trichothecene sesquiterpenes, verrucarin L acetate (VA, **92**) and roridin E (**93**), which were isolated from *Ficus fistulosa* Reinw. ex Bl. and *R. decursiva*, respectively [43]. Both trichothecenes showed the capability of killing the *P. falciparum* parasites at very low concentrations, but only VA demonstrated a good selective index (SI) (Table 4). Additional studies of structurally related or chemically modified trichothecenes might lead to more potent antimalarial compounds with greater selectivity indices.

The CHCl_3_-soluble extract of the stem of *Nauclea orientalis* (L.) L. (Rubiaceae; common name: Khan Leuang) collected in Laos also showed an in vitro inhibitory effect on the D6 and W2 clones of *P. falciparum* with IC_50_ values of 3 and 6 μg/mL, respectively [46]. Bioassay-guided fractionation of the antimalarial-active CHCl_3_ extract of the dried stem resulted in the isolation of two novel compounds, as well as six known compounds, four of them (**94**–**97**) showed moderate in vitro activities against *P. falciparum*. 

*Grewia bilamellata* Gagnep. (Tiliaceae) was found to be another promising lead in an anti-*P. falciparum* screening study. Bioassay-directed fractionation led to the isolation of 12 compounds from a sample of the dried leaves, twigs, and stems of this plant [45]. Five of the compounds showed varying degrees of in vitro antimalarial activity (**98**–**102**). 

*Gongronema napalense* (Wall.) Decne. (Asclepiadaceae) (synonym: *Gymnema napalense* Wall.), known as “Kheuang nguan mu” in Laos, is used locally in combination with one other species to treat polio, and this plant had also been previously reported for the treatment of leucorrhea, blennorrhea, and traumatic injury [49,51]. Bioassay-guided fractionation of the CHCL_3_ extract of this plant led to the isolation of a new steroidal glycoside, gongroneside A (**103**), with an IC_50_ value of 1.60 and 1.39 μM against the *P. falciparum* D6 and W2 clones, respectively [49]. 

*Diospyros quaesita* Thw. (Ebenaceae), locally known as “Muang kout” in Laos, was found to be a promising lead in the anti-*P. falciparum* bioassay. Antimalarial bioassay-directed fractionation of the CHCl_3_ extract led to the isolation of seven compounds, including one active compound, betulinic acid 3-caffeate (**104**) [50]. 

The stem sample of *Rourea minor* (Gaertn.) Alston. (Connaraceae), known as ‘‘KhuaMa Vo’’ and a decoction used locally to treat dengue fever [44], showed in vitro inhibitory effect on *P. falciparum*. Bioassay-directed fractionation of the antimalarial active CHCl_3_ extract of the dried stems of *R. minor* led to isolation of two glycosides and five known compounds. Three compounds (**105**–**107**) showed weak in vitro activities against *P. falciparum*.

## 3. Discussion and Conclusions

The UIC-ICBG project over 14-years periods (1998–2012), plus the subsequent continuous efforts carried out by researchers in Hong Kong, has resulted in the generation of a large database of information for the discovery of bioactive agents. A web-based “*Atlas of Seed Plants of Cuc Phuong National Park*” presently contains all of the 1926 species of angiosperms collected through the ICBG program [52]. Through the extensive biological and chemical studies of the active extracts and fraction, the ICBG team has isolated approximately 300 compounds of various chemical structures, from more than 30 active leads, including some highly active compounds and series of novel bioactive phytochemicals. The UIC-ICBG researchers further synthesized a library of derivatives of a number of active compounds and analyzed the structure-activity relationship. These results are expected to provide leads for further drug development. However, as the molecular targets and mechanisms of action of the active natural products are still unknown, continuing research of these lead compounds need to be carried out in order to develop them as future therapeutic drugs.

During the 14 years’ efforts of the UIC ICBG, they evaluated several thousand plant extracts against cancer, HIV, TB, malarial and bird flu virus and resulted in the identification of at least 100 bioactive compounds. However, a large number of the active plant leads have not been studied phytochemically. Some of the plant extracts have demonstrated potent bioactivity. For example, among the anticancer plant leads, the number of the active plant leads that showed cell killing activity with IC_50_ values of less than 5 μg/mL totaled 140, and the number with IC_50_ values of less than 1 μg/mL is 44. Among the antimalarial plant leads, the number of the active plant extracts that showed anti-*P. falciparum* activity with IC_50_ values within 5 μg/mL is 33, and the number with IC_50_ values within 1 μg/mL is 10. Thus, we believe that further exploration of these phytochemically uninvestigated active plant leads will produce a large number of novel and active compounds, which are considered as a valuable asset of the 14 years’ extensive research and achievements of the UIC based ICBG program.

## Figures and Tables

**Table 1:** The cell killing activity of compounds **1**–**34**.

No.	Compound Name	Bioactivity: IC_50_ (µM)						Plant Origin	Ref.
		KB	Lu-1	Col-2	LNCaP	MCF-7	HUVEC		
**1**	Miliusol	1.2	1.6	1.4	1.8	3.1	1.3	*Mliusa sinensis*	[9]
**2**	Miliusate	1.2	2.0	1.6	3.2	3.6	2.9	*M. sinensis*	[9]
**3**	Miliusane I	1.4	2.9	2.9	5.1	2.2	1.8	*M. sinensis*	[9]
**4**	Miliusane II	5.5	5.8	9.4	19.6	21.3	6.6	*M. sinensis*	[9]
**5**	Miliusane III	1.2	4.8	4.3	5.1	2.6	-	*M. sinensis*	[9]
**6**	Miliusane IV	32.2	60.4	38.5	>62.0	15.8	-	*M. sinensis*	[9]
**7**	Miliusane V	>55.0	>55.0	>55.0	>55.0	>55.0	-	*M. sinensis*	[9]
**8**	Miliusane VI	4.0	6.6	4.2	5.3	4.8	-	*M. sinensis*	[9]
**9**	Miliusane VII	5.8	6.2	3.7	5.8	6.1	-	*M. sinensis*	[9]
**10**	Miliusane VIII	47.4	63.6	33.4	43.4	26.4	>10.9	*M. sinensis*	[9]
**11**	Miliusane IX	>57.4	>57.4	46.0	>57.4	52.6	-	*M. sinensis*	[9]
**12/13**	Miliusane X/XI	5.2	21.4	8.0	29.6	5.0	-	*M. sinensis*	[9]
**14/15**	Miliusane XII/XIII	55.0	9.3	13.4	51.8	12.2	-	*M. sinensis*	[9]
**16/17**	Miliusane XIV/XV	5.3	7.5	5.4	27.6	10.1	-	*M. sinensis*	[9]
**18**	Miliusane XVI	6.1	19.9	3.9	6.1	6.4	-	*M. sinensis*	[9]
**19**	Miliusane XVII	6.7	14.9	9.5	24.0	11.0	-	*M. sinensis*	[9]
**20**	Miliusane XVIII	3.1	1.8	2.3	2.4	3.0	-	*M. sinensis*	[9]
**21**	Miliusane XIX	2.6	1.8	2.0	1.7	2.3	-	*M. sinensis*	[9]
**22**	Miliusane XX	>59.0	>59.0	>59.0	>59.0	>59.0	-	*M. sinensis*	[9]
**23**	Methoxyacetylmiliusol	16.4	25.3	15.6	>26.6	1.70	-		[9]
**24**	Asparacoside	4.8	4.2	5.4	10.1	-	4.1	*Asparagus cochinchinensis*	[10]
**25**	Asparacosins A	24.1	>45.0	>45.0	>45.0	-	>45.0	*A. cochinchinensis*	[10]
**26**	Asparacosins B	>39.6	>39.6	>39.6	>39.6	-	>39.6	*A. cochinchinensis*	[10]
**27**	3′′-methoxyasparenydiol	40.5	66.5	>67.5	>67.5	-	>67.5	*A. cochinchinensis*	[10]
**28**	Asparenydiol	8.5	70.1	>75.1	>75.1	-	>75.1	*A. cochinchinensis*	[10]
**29**	3′-hydroxy-4′-methoxy-4′-dehydroxynyasol	31.9	25.5	41.4	41.1	-	58.1	*A. cochinchinensis*	[10]
**30**	Nyasol	>79.3	>79.3	>79.3	>79.3	-	>79.3	*A. cochinchinensis*	[10]
**31**	3′′-methoxynyasol	31.9	15.9	22.3	23.4	-	23.7	*A. cochinchinensis*	[10]
**32**	1,3-bis-di-*p*-hydroxyphenyl-4-penten-1-one	>74.5	>74.5	>74.5	>74.5	-	>74.5	*A. cochinchinensis*	[10]
**33**	*trans*-coniferyl alcohol	>109.9	>109.9	>109.9	>109.9	-	>109.9	*A. cochinchinensis*	[10]
**34**	4′-demethyldesoxypodophyllotoxin	0.05	-	0.06	0.03	-	-	*Bursera tonkinensis*	[11]
	vinblastine	0.00037	0.11	0.0043	0.00061	0.0026	-		

**Table 2:** The anti-HIV activity of compounds **35**–**76**.

No.	Compound Name	IC_50_ (µM)	SI ^a^	Plant Origin	Ref.
**35**	Litseaverticillol A	21.4	2.6	*Litsea verticillata*	[14]
**36**	Litseaverticillol B	8.5–2.8	2.8–1.9	*L. verticillata*	[15]
**37**	Litseaverticillol C	30.3	2.4	*L. verticillata*	[15]
**38**	Litseaverticillol D	57.6	>1.0	*L. verticillata*	[15]
**39**	Litseaverticillol E	16.0	3.1	*L. verticillata*	[15]
**40/41**	Litseaverticillol F/G	45.2	1.7	*L. verticillata*	[15]
**42**	Litseaverticillol H	Toxic	-	*L. verticillata*	[15]
**43/44**	Litseaverticillol L/M	49.6	NT ^b^	*L. verticillata*	[20]
**45**	Isolitseane A	-	-	*L. verticillata*	[17]
**46**	Isolitseane B	38.1	3	*L. verticillata*	[17]
**47**	Isolitseane C	-	-	*L. verticillata*	[17]
**48**	Verticillatol	144.7	NT ^b^	*L. verticillata*	[18]
**49**	Litseagermacrane	27.5	2.3	*L. verticillata*	[15]
**50**	5-epieudesm-4(15)-ene-1β,6β-diol	73.1	NT ^b^	*L. verticillata*	[15]
**51**	Litseachromolaevane B	119.7	NT ^b^	*L. verticillata*	[15]
**52**	Oxyphyllenodiol B	54.6	NT ^b^	*L. verticillata*	[17]
**53**	1,2,3,4-tetrahydro-2,5-dimethyl-8-(1-methylethyl)-1,2-naphthalenediol	91.0	NT ^b^	*L. verticillata*	[17]
**54**	(+)-5′-demethoxyepiexcelsin	42.7	1.4	*L. verticillata*	[18]
**55**	3-*epi*-litsenolide D_2_	9.9	4	*L. verticillata*	[17]
**56**	Litseabutenolide	40.3	NT ^b^	*L. verticillata*	[17]
**57**	4-hydrixy-2-methylbut-2-enolide	129.8	NT ^b^	*L. verticillata*	[17]
**58**	Hydroxydihydrobovolide	122.7	NT ^b^	*L. verticillata*	[17]
**59**	Vaticinone	15.3	1.4	*Vatica cinerea*	[19]
**60**	*(23E)*-27-*nor*-3β-hydroxycycloart-23-en-25-one	21% inhibition @ 5.9 µM	-	*V. cinerea*	[19]
**61**	*(24E)*-3-oxo-lanosta-8,24-dien-26-oic acid	8.6	1.7	*V. cinerea*	[19]
**62**	Dammara-20,25-dien-3β,24-diol	6.1	2.3	*V. cinerea*	[19]
**63**	*(23E)*-dammara-20,23-dien-3β,25-diol	22.6	1.3	*V. cinerea*	[19]
**64**	Betulinic acid	32.5	1.1	*V. cinerea*	[19]
		3.1	5	*V. cinerea*	[21]
**65**	Betulin	13.8	1.4	*V. cinerea*	[19]
**66**	Betulonic acid	21.4	4.9	*V. cinerea*	[19]
**67**	Ursolic acid	14.7	1.1	*V. cinerea*	[19]
		14.4	1.0	*Strychnos vanprukii*	[21]
**68**	Pheophorbide *a*	2.5	>13	*Vatica cinerea*	[19]
**69**	1-hydroxy-cyclocolorenone	88.0	NT ^b^	*V. cinerea*	[19]
**70**	3β-*O*-*trans*-feruloylbetulinic acid	5.1	3.0	*Strychnos vanprukii*	[21]
**71**	3β-*O*-*cis*-feruloylbetulinic acid	11.1	2.0	*S. vanprukii*	[21]
**72**	3β-*O*-*cis*-coumaroylbetulinic acid	8.0	2.0	*S. vanprukii*	[21]
**73**	3β-*O*-*trans*-coumaroylbetulinic acid	5.6	3.0	*S. vanprukii*	[21]
**74**	3-(4-hydroxyphenyl)-1-(2,4,6-trimethoxyphenyl)-2-propen-1-one	<15.2	>1.6	*Vitex leptobotrys*	[22]
**75**	Tsugafolin	118	-	*V. leptobotrys*	[22]
**76**	Alpinetin	130	NT ^b^	*V. leptobotrys*	[22]
	3TC	0.29			

^a^ SI = selectivity index = CC_50_/IC_50_; ^b^ NT = n on-toxic at 20 μg/mL.

**Table 3:** The anti-TB activity of compounds **77**–**88**.

No.	Compound Name	MIC (µM)	Plant Origin	Ref.
**77**	(−) *Z*-9-octadecen-4-olid	5.3	*Micromelum hirsutum*	[35]
**78**	Micromeline	112.9	*M. hirsutum*	[35]
**79**	Lansine	59.3	*M. hirsutum*	[35]
**80**	3-formylcarbazole	216.9	*M. hirsutum*	[35]
**81**	3-formyl-6-methoxycarbazole	69.3	*M. hirsutum*	[35]
**82**	5-(8*Z*-heptadecenyl) resorcinol	34.4	*Ardisia gigantifolia*	[37]
**83**	5-(8*Z*-pentadecenyl) resorcinol	79.2	*A. gigantifolia*	[37]
**84**	-	42.0	*A. gigantifolia*	[37]
**85**	Bonianic acids A	34.9	*Radermachera boniana*	[36]
**86**	Bonianic acids B	9.9	*R. boniana*	[36]
**87**	Ergosterol peroxide	3.5	*R. boniana*	[36]
**88**	8-hydroxy-6-methoxy-pentylisocoumarin	153.4	*Xylosma longifolia*	[38]
	Rifampin	0.049		

**Table 4:** The antimalarial activity of compounds **89**–**107**.

No.	Compound Name	KB	D6		W2		Plant Origin	Ref.
		ED_50_ (µM)	IC_50_ (µM)	SI ^a^	IC_50_ (µM)	SI ^a^		
**89**	Polysyphorin	4.8	1.0	5.0	0.9	6.0	*Raphidophora decursiva*	[48]
**90**	Rhaphidecurperoxin	13.1	1.8	0.7	1.37	1.0	*R. decursiva*	[48]
**91**	Decursivine	-	11.3	-	12.7	-	*R. decursiva*	[47]
**92**	Verrucarin L acetate	0.17	0.0011	158.0	0.0012	135.0	*R. decursiva*	[43]
**93**	Roridin E	0.00041	0.00039	1	0.0012	0.4	*R. decursiva*	[43]
**94**	Naucleaorine	38.0	6.9	5.5	8.0	4.8	*Nauclea orientalis*	[46]
**95**	Epimethoxynaucleaorine	>37.9	12.4	>3.1	13.2	>2.9	*N. orientalis*	[46]
**96**	3α,23-dihydroxyurs-12-en-28-oic acid	>42.2	9.7	>4.4	12.7	>3.3	*N. orientalis*	[46]
**97**	Oleanolic acid	46.0	4.6	10	5.1	9.1	*N. orientalis*	[46]
**98**	3α,20-lupandiol	>90.0	19.8	>4.5	19.1	>4.7	*Grewia bilamellata*	[45]
**99**	Grewin	>107.5	11.2	>9.6	5.5	>19.7	*G. bilamellata*	[45]
**100**	Nitidanin	>99.0	21.2	>4.6	18.4	>5.4	*G. bilamellata*	[45]
**101**	2*R*,3β-dihydroxyolean-12-en-28-oic acid	51.5	21.1	2.4	8.6	5.9	*G. bilamellata*	[45]
**102**	2,6-dimethoxy-1-acetonylquinol	169.0	42.2	4.0	23.0	7.3	*G. bilamellata*	[45]
**103**	Gongroneside A	>13.7	1.6	>8.5	1.4	>9.8	*Gongronema napalense*	[49]
**104**	Betulinic acid 3-caffeate	4.0	1.4	2.9	1.0	4.0	*Diospyros quaesita*	[50]
**105**	Rourinoside	>35.9	3.7	>9.5	2.1	>16.7	*Rourea minor*	[44]
**106**	Rouremin	>25.5	5.1	>5.0	4.5	>5.7	*R. minor*	[44]
**107**	1-(26-hydroxyhexacosanoyl)-glycerol	>45.2	9.5	>4.3	12.7	>3.2	*R. minor*	[44]
	Artemisinin	>70	0.007	>10,000	0.007	>10,000		

^a^ SI = Selectivity Index = ED_50_ KB/IC_50_ *P. falciparum*.
